# STAR Duodecim eHealth Tool to Recognize Chronic Disease Risk Factors and Change Unhealthy Lifestyle Choices Among the Long-Term Unemployed: Protocol for a Mixed Methods Validation Study

**DOI:** 10.2196/27668

**Published:** 2021-06-01

**Authors:** Henna Kuhlberg, Sari Kujala, Iiris Hörhammer, Tuomas Koskela

**Affiliations:** 1 Faculty of Medicine and Health Technology Tampere University Tampere Finland; 2 Department of Computer Science Aalto University Helsinki Finland; 3 Department of Industrial Engineering and Management Aalto University Helsinki Finland; 4 Department of General Practice Faculty of Medicine and Health Technology Tampere University Tampere Finland; 5 Center of General Practice Tampere University Hospital Tampere Finland

**Keywords:** eHealth, risk assessment, long-term unemployed, expected age of death, online intervention, risk factors, chronic illnesses, primary prevention, online health check, long-term, multimorbidity, health care services, examination, evaluation, lifestyle

## Abstract

**Background:**

Lifestyle choices and socioeconomic status have a significant impact on the expected onset of diseases, age of death, and risk factors concerning long-term illnesses and morbidity. STAR is an online health examination tool, which gives users a report that includes an evaluation of their life expectancy and an estimated risk for developing common long-term illnesses based on questions about health, characteristics, lifestyle, and quality of life.

**Objective:**

The goals of this study are to (1) review the capacity of STAR to recognize morbidity risks in comparison to a traditional nurse-led health examination and patient-reported health challenges; (2) evaluate the user experience and usability of STAR; and (3) assess the potential impact of STAR on the health confidence and motivation of patients to make healthier lifestyle choices.

**Methods:**

This mixed methods validation study will consist of a quantitative part (questionnaires) and a qualitative part (phone interviews and open-ended questions from the questionnaires). The participants will include 100 long-term unemployed individuals attending a health check for the unemployed. The participants will be recruited from three Finnish public health centers in Espoo, Hämeenlinna, and Tampere. At the health centers, the participants will use STAR and attend a nurse’s health check. Surveys with multiple-choice and open-ended questions will be collected from the participants, the nurse, and a study assistant. The questionnaires include questions about the participant’s background and health challenges from the patient and nurse points of view, as well as questions about how well the health challenges matched the STAR report. The questionnaires also gather data about user experience, health confidence, and usability of STAR. A study assistant will fill out an observer’s form containing questions about use time and possible problems encountered while using STAR. A sample of the unemployed participants will be interviewed by telephone subsequently. For the quantitative data, descriptive statistics and a reliability analysis will be performed, and mean sum scores will be computed for the study variables. Thematic analysis of the qualitative data will be performed.

**Results:**

This study was approved by the Ethics Committee of the Expert Responsibility Area of Tampere University Hospital in June 2020 (ETL Code R20067). Data collection will begin in June 2021 and will take approximately 3-6 months.

**Conclusions:**

Online health examinations can improve the effectiveness of primary prevention in health care by supporting efficient evidence-based morbidity risk estimation and motivating patients to change unhealthy behaviors. A multimethod approach is used to allow for assessment of the tool’s usefulness from the points of view of both professionals and patients. This study will further provide a rich understanding of how the tool can be used as part of routine health checks, and how and why the tool may or may not motivate users for making healthier lifestyle choices.

**International Registered Report Identifier (IRRID):**

PRR1-10.2196/27668

## Introduction

### Background

Long-term illnesses and multimorbidity have become more common, thus reducing quality of life and increasing the demand for health care services [1,2]. Lifestyle choices have a significant impact on the expected onset of diseases, age of death, risk factors concerning long-term illnesses, and multimorbidity [1,3-5]. Preventable lifestyle-related risk factors affecting chronic morbidity and mortality have been recognized, most notably smoking, the harmful use of alcohol, physical inactivity, and an unhealthy diet [5,6].

eHealth uses digital information and communication technologies for health, demonstrating growing potential to make health services more accessible, efficient, and cost-effective [7,8]. eHealth interventions aimed at assessing lifestyle-related risk factors could be one possible way to improve primary prevention in public health care [9-11]. An online health-related risk behavior intervention can be used to acquire clinical health behavior information, help health care professionals with standardized risk estimation, and motivate the patient to change unhealthy behaviors [9]. Web-based interventions focusing on health behavior–related risks have been reported to have an overall positive effect on the user’s health, resulting in positive behavior changes [11-13]. Assessing multiple lifestyle-related risks at the same time provides an opportunity to review one’s health comprehensively and target multiple health-related risk behaviors simultaneously [9]. Such interventions have been received well by patients compared to interventions targeting only one health-related behavior [9,12-14].

The long-term unemployed comprise a particular subgroup that could greatly benefit from such interventions [15]. Long-term unemployment is linked to greater than average morbidity, earlier expected age of death, and increased risk of mortality [15,16]. Long-term unemployment is defined as having been unemployed for 12 months or more [17]. The duration of unemployment increases the burden of disease [18]. Unemployment also affects self-assessed health negatively, and the strongest association is found in people with a lower socioeconomic status, weak social networks, and health-related reasons for unemployment [19]. There have been studies on online health checks and internet-based risk assessments of subgroups such as the employed, but there have been few studies focusing on online health checks for the unemployed [20].

There has been growing interest in studying the use of eHealth tools in preventing and treating chronic illnesses [21]. Although there are also some mixed results, positive evidence has been found on the effect of online health interventions in several different fields [8-14,21-23]. A systematic review of mobile and online health lifestyle interventions found them to have an up to 12-month positive effect on health [23]. A meta-analysis of computer-tailored interventions for health behavior change [11] found a clinically and statistically significant effect on all four health behaviors of focus. However, the effect of the intervention decreased over time; thus, more studies about the sustained effect are needed [11,23].

There is a huge variety of health risk calculators available online. A systematic review of online cardiovascular disease risk calculators found wide variation in risk assessment models, risk presentation, and results [24]. The study also found the risk calculators to have overall poor actionability, and that the available risk calculators often lack clinical validity. The information provided by risk calculators can help health care professionals to identify correct risk categories more accurately and also improve the likelihood of prescribing medicine to high-risk patients, thereby helping with decision-making [25]. A systematic review of decision aids used in clinical encounters reported that clinical decision systems improve satisfaction with medical decision-making; furthermore, clinicians found the information provided to be helpful [26].

An individual’s perception of the likelihood and severity of a disease is a critical determinant of health behavior [27,28]. In addition to risk perceptions, a systematic literature review reported that motivation, support, and feedback are the most influential factors in changing health-related behavior with eHealth tools [29]. The lack of these same factors is also cited most often as a barrier to use. Goal-setting and self-regulation, rewards, user-friendliness, accessibility, and access to remote help are also mentioned as facilitating factors, while lack of resources or priority, negative support, lack of information, issues with technology, and sociodemographic barriers are listed as barriers of use. A scoping review of the usability and utility of eHealth for physical activity counseling in primary health care centers also found technical problems and the complexity of programs to be notable usability barriers to eHealth [30].

A systematic review of sociodemographic factors influencing the use of eHealth in people with chronic diseases found that the people who could benefit the most from eHealth interventions are usually not using them. The authors suggested tailoring the interventions to be more personal, making them more accessible, and using them to complement health care [31]. Another systematic review suggested making the digital health interventions more visible to the public, incorporating communication with health care professionals and people with similar health problems, and involving clinical organizations or clinicians to promote and validate digital health interventions [32]. Similar recommendations were also made in other studies [33,34].

### Intervention

The STAR Duodecim Health Check and Coaching Program (hereafter, STAR) is a general online health examination developed by Duodecim Publishing Company Ltd and the Finnish Institute of Health and Welfare [35-37]. The abbreviation STAR comes from the Finnish words for an online health check. STAR assesses the user’s health, lifestyle, and mental well-being, and provides users a report including an evaluation of life expectancy and the estimated risk of developing the following common long-term illnesses: coronary heart disease, stroke, diabetes, and dementia. The report is based on approximately 40 questions regarding the user’s health, demographic characteristics, lifestyle, and quality of life. The report also has suggestions on how to change to a healthier lifestyle, reduce the multimorbidity and long-term illness risks, and lengthen life expectancy. STAR and its report are further described in Multimedia Appendix 1. The life expectancy evaluation and the risk evaluations are based on previous Finnish studies, namely the Finriski, Autoklinikka, and Minisuomi studies [35,38-40].

Previous studies of STAR have mainly focused on creating a persuasive system design [41-43]. One of these studies established that consumers found the health feedback of STAR and its online coaching program to be too general; they instead desired more personalized feedback [41]. In general, the consumers had a favorable impression of STAR and there were no concerns with its credibility. In addition, a 2-year follow-up study on STAR’s online training programs was performed in Finland, showing moderate positive improvement on stress, gratitude, and confidence in the future, although the effect decreased over the 2-year follow-up period [43]. The study included over 40,000 participants, which is a sign of interest in eHealth and its possibilities, although only 15% of the participants continued to the 2-year follow-up point.

STAR can be used either as a self-led program or integrated to a health care system. Duodecim’s STAR Pro allows medical professionals to view their patients’ STAR reports, which can be used, for example, to help with decision-making and as a useful means to review the patient’s health and lifestyle choices. The user, be it the patient or the health care professional, can use their email to log in to the website. The service is cloud-based. STAR is available to approximately 2 million Finns as part of public health care service choices in selected areas of Finland, including in Vantaa, Salo, Seinäjoki, and the Keski-Uudenmaa municipal consortium [37,44-47]. STAR also has online training programs for maintaining health. There are currently six different themes: exercise, healthy nutrition, sleep, weight control, strengthening mental resources, and everyday stress control. The STAR report recommends these programs to the user based on the answers given. After using STAR for the first time, users can log in again with their email address to perform a new health check, follow their progress, or start the online training programs.

### Study Objectives

The aim of this study is to help validate STAR as a risk assessment–based online health examination. The primary objectives of this study are to review STAR’s ability to recognize health challenges among the long-term unemployed and to assess the potential of STAR to make a positive impact on the lifestyle choices of the unemployed.

The specific goals of this study are to: (1) review the capacity of STAR to recognize morbidity risks in comparison with a traditional nurse-led health examination and patient-reported health challenges; (2) evaluate the user experience and usability of STAR; and (3) assess the potential impact of STAR on the health confidence and motivation of patients to make healthier lifestyle choices.

## Methods

### Recruitment

People who have been unemployed for at least 12 months, are over 18 years old, and are participating in a health check for long-term unemployed people will be recruited for this study. Finland has a public health care system organized and financed by municipalities, and every resident is entitled to receive social, health, and medical services [48,49]. Therefore, the municipalities are obligated to organize health checks for the unemployed. The purpose of these health checks is to advance the health, and the ability to function and work of unemployed people [50]. The initiative can come from the unemployed person, unemployment services, or the municipal social welfare administration.

The goal is to recruit 100 participants in total. The recruitment will take place at three Finnish public health centers in Espoo, Hämeenlinna, and Tampere. Espoo and Tampere are the second and the third largest cities in Finland, with a population of 290,000 and 240,000, respectively [51]; Hämeenlinna is a slightly smaller city, with a population of 68,000. When the clients are booking their appointment, they will be informed about this study and the opportunity to participate. The participants will be asked to provide information about their cholesterol, blood pressure, and waist measurement at the appointment. A study assistant will be waiting for them at the local clinic where the health check is performed. The study assistant will ensure that the informed consent of the participants is obtained by giving them an information sheet on the study and answering any possible questions about the study. The consent form has an additional field for the participant’s phone number, which will be used to perform a telephone interview 2 weeks after the essential health check portion of the study.

### Study Setting

**Figure 1:**
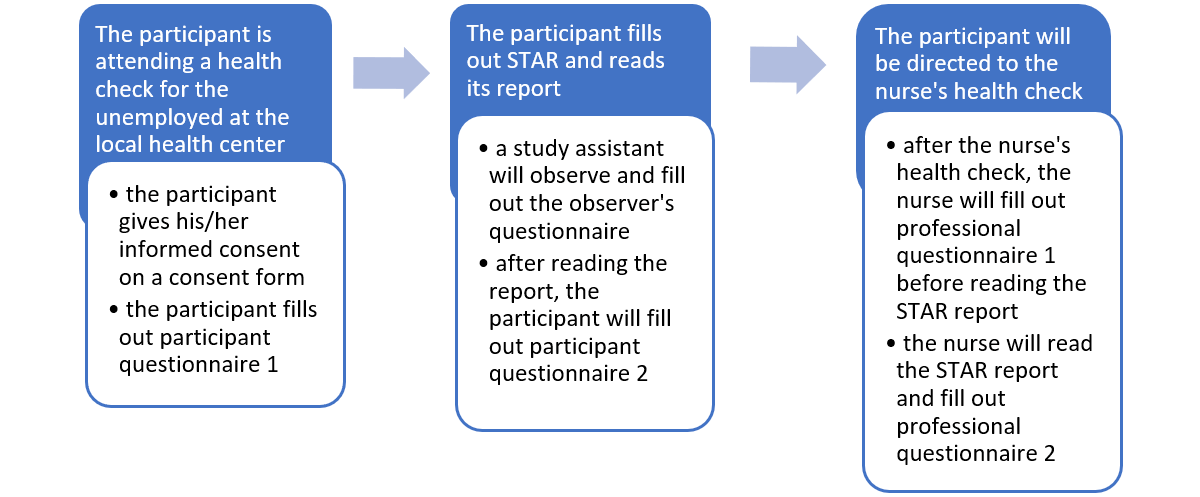
Flow of the study.

The participants will start their participation in the study after signing the consent form (Figure 1). The study assistant will give them the first questionnaire, which contains questions about the participant’s background information. After filling out the first questionnaire, the participants will be asked to perform the STAR online health examination. The study assistant will open STAR via a study email link and fill out an observer’s form while observing the participant using STAR. The form has questions about the time taken to fill out STAR and read its report, and about the possible difficulties the participant experienced while using it. After filling out STAR and reading the report, the participants will fill out a second questionnaire concerning the user experience, their health confidence, how well they think STAR recognized their health challenges, and whether they would recommend STAR to a friend.

The participants will also attend a general health check in the nurse’s office. The nurse’s health check takes approximately 60 minutes to complete, including an anamnesis form; interview; and filling out evaluation forms for diabetes risk, alcohol use (audit), and depression (BDI-test). Blood pressure, weight, height, waist circumference, and BMI will also be measured. Before the health check, patients are given a referral for laboratory tests to check their blood sugar and lipids so that the results can be discussed at the health check. The health checks will be adjusted to the patient’s personal needs. After the health check and before reading the STAR report, the nurse will fill out a two-part questionnaire regarding each participant’s health challenges and STAR report. After filling out the first part, the nurse will read the participant’s STAR report on the STAR Pro view and then fill out the second part, which includes questions about STAR’s ability to recognize the participant’s health challenges and whether the nurse found the STAR report to be useful from a medical professional’s point of view.

**Figure 2:**
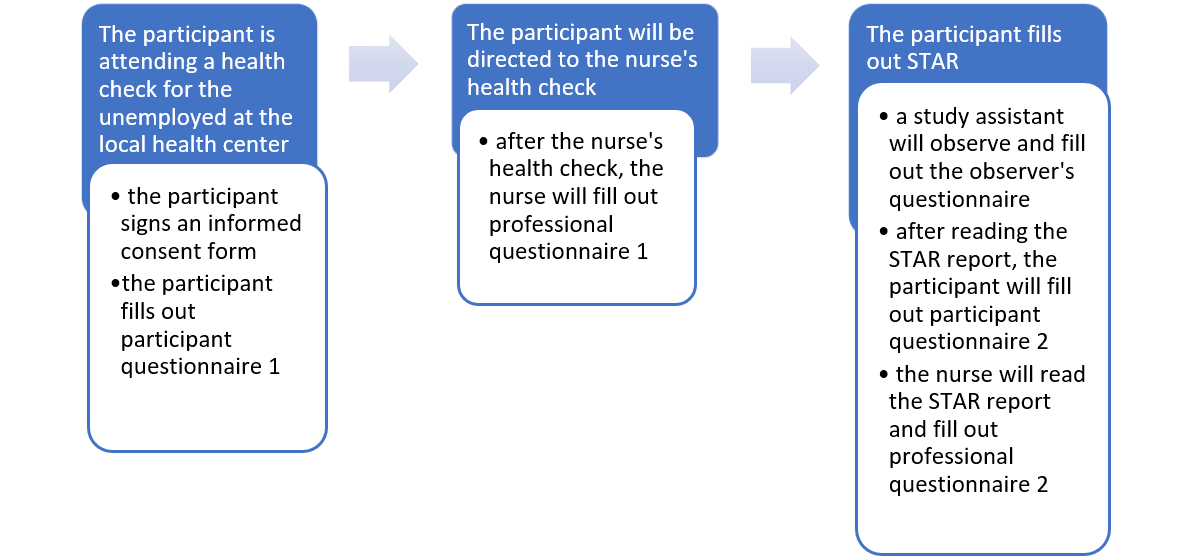
Counterbalanced situation.

To balance STAR’s effect on the nurse’s health check, half of the health checks will be counterbalanced; that is, the order of the STAR and the nurse’s health check will be reversed after every 10 check-ups (Figure 2). The forms will be filled out accordingly. The study protocol will be piloted for 1 day at a health center before starting the data collection.

### Phone Interviews

A sample of patients who grant permission for a phone interview will be interviewed approximately 2 weeks after the health check. Semistructured interviews will be used to ask more open**-**ended questions about using STAR and the online training programs (an open-ended question is a question that cannot be answered with a simple “yes” or “no,” and requires longer answers to explain one’s point of view). The questions focus on the experiences of using the tools, and their potential to support the respondents’ healthy lifestyle choices and motivation to manage their own health. The interviews will last from 30 minutes to 1 hour, and will be audio-recorded using Teams and transcribed for analysis.

### Questionnaires

There are five questionnaires in total for this study: the participant questionnaires (parts 1 and 2), study assistant’s questionnaire, and nurse’s questionnaires (parts 1 and 2). The questionnaires include questions on the participant’s background and the most significant health challenges from their own and the nurse’s point of view, as well as on how well the health challenges matched the STAR report. The questionnaires also gather data on the user experience, usability, and health confidence. The first parts of the forms will be filled out before the participant uses STAR and before the nurse reads the STAR report. The second parts will be filled out after reading the STAR report. A study assistant will also fill out an observer’s form containing questions about the time used and possible problems encountered while using STAR. The questionnaires are presented in Multimedia Appendix 2.

Usability will be measured by asking questions based on the Usability Metric for User Experience [52]. The client’s health confidence will be measured with questions based on the Health Confidence Score [53].

### Analysis Methods

The data will be analyzed with quantitative and qualitative methods.

The risk assessments and health recommendations given by the STAR report will be reviewed by determining the most crucial health risks specified by STAR among unemployed participants. The three health challenges determined by the client and the nurse’s health check will be compared to the STAR report by calculating the matching percentages and their confidence intervals. These health challenges will first be classified into corresponding categories so that they can be compared to the STAR report. The new health challenges STAR found and any health challenges missed will be analyzed. The user experience and usability of STAR will be analyzed by assessing the responses from surveys.

Data from the phone interviews and open-ended questions of the survey will be analyzed using qualitative theme analysis. All phone interviews will be performed by two research group members (SK, IH), who have previous experience regarding phone interviews. The phone interviews will be recorded and transcribed verbatim. We will start to read and reread data to become familiar with what the data entail, paying specific attention to patterns that occur. The results of the first phase will create preliminary codes. The coding will be performed by all four group members. Three of the coders have previous experience in coding. After initial coding, a meeting of the coders will be held to discuss codes and categories. The final list of codes will be the result of consensus among all members of the coding group. The next steps of the thematic analysis are: (1) combining codes to themes, (2) interpretation of the codes, and (3) explanation of the contribution of each theme to understanding STAR’s usability and impact on lifestyle choices and motivation.

### Ethical Approval

This study was approved by the Ethics Committee of the Expert Responsibility Area of Tampere University Hospital in June 2020 (ETL Code R20067), and commenced at the start of 2021. The results of the study are expected to be published at the end of 2021.

## Results

The data collection will begin in June 2021. The data collection will take approximately 3-6 months. 

## Discussion

### Limitations and Potential Concerns

The limitations and concerns of this study involve the recruitment of the participants among the unemployed, the potential selection bias toward those better able to benefit from the intervention, and the potentially varying interpretations of “health challenges” among the unemployed and study nurses. The study material will be slow to gather, because the health centers have a loose schedule for performing health checks for long-term unemployed clients. There will be a limited number of nurses working on the health checks at the same time, and there is no certainty that the few unemployed people who are participating in a health check will agree to participate in the study. Motivating the unemployed to participate in this study may turn out to be difficult due to their individual and complex situations. However, they could be more flexible with the additional time the study takes when compared to those in the working population. Additionally, the COVID-19 pandemic has created new challenges and restrictions for public health care, which may affect the data gathering of this study. Moreover, due to social distancing, it may be harder to recruit participants, because it has been advised to avoid any unnecessary contacts, and some may categorize a general health check as such. We will take care of all appropriate COVID-19 safety measures to make the situation as safe as possible for all parties involved.

The use of a digital tool as an intervention may have an effect on the participants. There is a concern of selection bias, because people who are comfortable with digital tools will more readily agree to participate compared with those who are not familiar with such technology. People who struggle with computers and technology may refuse to participate, even though they could provide important knowledge about usability and user experience for the study. It has been reported that the users of eHealth interventions are more likely to be highly educated and have a healthier lifestyle than average, while those who could benefit the most are not using them [31,33].

The health challenges determined by the client and the nurse could differ significantly from those highlighted in the STAR report because every individual can interpret the terms differently. We will try to solve this discrepancy by categorizing the health challenges; however, it is difficult to foresee how the health challenges determined by the participant, the nurse, and STAR will correspond.

### Conclusions

As part of public health care, eHealth technologies have significant potential in solving problems regarding the current growing trend in long-term illness morbidity and multimorbidity [8-10]. Although studies on online health behavior interventions are increasing, there are few studies on online health checks and online risk calculators as part of public health care and in the area of recognizing multiple different lifestyle behavior risk factors. This exploratory research will contribute to this field as a starting point on the fieldstream of online general health check research by examining the mechanisms through which an online health check integrated into public health care may enhance the recognition of chronic disease risk and impact the health behavior of the unemployed.

## Appendix

Multimedia Appendix 1 The content of Duodecim’s STAR.

Multimedia Appendix 2 The questionnaires used in the study.
