# Supplementary material for: STAR Duodecim eHealth Tool to Recognize Chronic Disease Risk Factors and Change Unhealthy Lifestyle Choices Among the Long-Term Unemployed: Protocol for a Mixed Methods Validation Study
Source: JMIR Res Protoc. 2021 Jun 1;10(6):e27668. doi: 10.2196/27668 (PMC8207252; doi:10.2196/27668)
Supplement: Multimedia Appendix 1 [file resprot_v10i6e27668_app1.docx]

Appendix 1

The content of Duodecim’s STAR

***Collecting information from the user***

STAR asks the user approximately 40 questions. The first category includes characteristics like sex, age, height, weight, waist measurement, and years of education. The second category is health information, where STAR asks for the user’s total cholesterol, HDL cholesterol, and systolic and diastolic blood pressure. There are also questions about blood pressure medication, history of family morbidity, and the user’s existing health conditions in terms of the most common long-term diseases, like coronary heart disease, stroke, asthma, diabetes, and musculoskeletal disorders. Oral hygiene is also included. The third category is intoxicant use, where STAR asks the client about tobacco and alcohol use. The fourth category is nutrition. The questions ask about the user’s vegetable, fruit, berry, whole grain, and fish intake. The fifth category is exercise and free time, which contains questions about the user’s weekly physical activity and hobbies. The sixth category is sleep, stress, mental welfare, and working capacity. There are questions about the user’s sleeping hours, daily stress, life goals, future, ability to live in the moment, appreciation of life, and estimated ability to work in the current occupation in the future. The seventh and final category is family and relationships, which asks questions about the user’s close relationships and family. There are five mandatory questions the user must answer to continue.

***The report***

The STAR report gives the user a graphic presentation of his/her estimated life expectancy and number of healthy years, and an evaluation of how much the user could affect them by making better lifestyle choices. It also specifies the user’s most important long-term disease risks in graphic form. STAR sorts the answers into three different categories: 1. “Necessary to review”; 2. “Check and keep in mind”; and 3. “Great, keep up the good work!” The corresponding categories also have links to Finnish health databases for more information on the subject, for example the Terveyskirjasto.fi health library. After reviewing the answers, STAR gives the user recommendations for improving health behavior to reduce morbidity risks, increase the number of healthy years, and lengthen life expectancy. They are separated into two categories: 1. “Start with these!” and 2. “You can also work on these.” The first category focuses on the things that are necessary to improve the user’s health and have a major impact on morbidity risks and life expectancy. The second category focuses on the things that the user could work on to get a better result, but the impact on health is smaller. In addition, the report gives the user recommendations for online training programs according to the user’s health needs.
