# Supplementary material for: STAR Duodecim eHealth Tool to Recognize Chronic Disease Risk Factors and Change Unhealthy Lifestyle Choices Among the Long-Term Unemployed: Protocol for a Mixed Methods Validation Study
Source: JMIR Res Protoc. 2021 Jun 1;10(6):e27668. doi: 10.2196/27668 (PMC8207252; doi:10.2196/27668)
Supplement: Multimedia Appendix 2 [file resprot_v10i6e27668_app2.docx]

Appendix 2

The questionnaires used in the study

**The** **participant’s (unemployed person’s) questionnaire, part 1**

The participant’s questionnaire, part 1 is filled before using STAR. It gathers mainly quantitative background information from the participant. It includes questions on the respondent’s age, sex, length of unemployment, occupation before unemployment, level of education, and long-term illnesses. In addition, there is an open question about the participant’s three most significant health challenges.

**The study assistant’s questionnaire**

The study assistant’s questionnaire is filled by the study assistant while he/she observes the participant using STAR. It contains the following questions:

1. How long did it take for the participant to fill the online health check?

2. In your estimation, how easy was it for the participant to use the online health check? Answers are on a graded scale of 1 (very difficult) to 5 (very easy).

3. Did the participant have any severe problems during the online health check that he/she could not handle without help? The observer writes down the number of problems and records their nature in an open field.

4. Did the participant have any significant problems during the online health check that disturbed or slowed down his/her progress? The observer writes down the number of problems and records their nature in an open field.

5. Did the participant have any small cosmetic problems while using the health check, which disturbed or slowed down his/her progress? The observer writes down the number of problems and records their nature in an open field.

6. Are there any other observations regarding the online health check? There is an open field for the answer.

7. Did the online health check evoke any visible emotions in the participant? (Yes/No) If the answer is “yes,” there is an open follow-up question: What emotions, and for what reason?

8. Did the participant read the online health check’s report? (Yes/No) If the answer is “yes,” there is an open follow-up question: How long did it take for the participant to read the report?

**The participant’s (unemployed person’s) questionnaire, part 2**

The participant’s questionnaire, part 2 is filled by the participant after filling STAR and having the opportunity to read its report. It contains the following questions and statements:

1. Were the questions and terms of the online health check easy to understand? (Yes/No) If the answer is “no,” there are open follow-up questions: What questions or terms were unclear? How could they be improved?

Numbers 2-7 are statements that are answered on the scale of 1 (strongly disagree) to 5 (strongly agree).

2. The online health check and its report are useful to me.

3. Using the online health check is frustrating.

4. The online health check is easy to use.

5. The online health check’s report gave me new information about my health.

6. The online health check’s report gave me new information about how I could influence my own health.

7. The online health check’s report gave me new information about how I could get help to take care of my health.

8. Did the online health check miss something important regarding your health? (Yes/No) If the answer is “yes,” there is an open field to describe what it missed.

9. What did you think about the life expectancy and the morbidity risks presented in the report? There is an open field for the answer.

10. How likely is it that you would recommend this service to someone you know? The answer is on a scale of 1 (very unlikely) to 10 (very likely), and there is an open field to explain why.

**The nurse’s questionnaire, part 1**

The nurse’s questionnaire, part 1 is filled by the nurse after the participant’s health check and before seeing the STAR report. It has a question about the participant’s three most significant health challenges based on the nurse’s health check.

**The nurse’s questionnaire, part 2**

The nurse’s questionnaire, part 2 is filled by the nurse after reading the STAR report. It has the following questions:

1. Did STAR notice something essential about the patient’s health that did not come up in the nurse’s health check? (Yes/No) If the answer is “yes,” the respondent is asked to describe what it was in an open field.

2. Did STAR miss something essential regarding the patient’s health that you noticed? (Yes/No) If the answer is “yes,” the respondent is asked to describe what it was in an open field.

3. How useful do you think the STAR report was for your work? The answer is on a scale of 1 (completely useless) to 5 (extremely useful). There is also an open follow-up question: What are the benefits and challenges of the STAR report?

4. How easy to use was STAR’s PRO view? The answer is on a scale of 1 (very difficult to use) to 5 (very easy to use). There is also an open follow-up question: Do you have any suggestions for STAR’s PRO view?
